# Synthesis and crystal structures of [Ph_3_PCH_2_PPh_3_]I_2_ di­chloro­methane disolvate and [Ph_3_PCH_2_PPh_3_](BI_4_)_2_

**DOI:** 10.1107/S2056989017010295

**Published:** 2017-07-28

**Authors:** Rakesh Ganguly, Violeta Jevtovic

**Affiliations:** a Division of Chemistry & Biological Chemistry, SPMS-CBC-01-18D, Nanyang Technological University, 21 Nanyang Link, 637371, Singapore; b Department of Biological Sciences and Chemistry, College of Arts and Sciences, University of Nizwa, Sultanate of Oman

**Keywords:** crystal structure, carbodi­phospho­rane, C—H⋯I hydrogen bonding

## Abstract

Reaction of BI_3_ with carbodi­phospho­rane, C(PPh_3_)_2_, gives a mixture of the dicationic compounds, methyl­enebis(tri­phenyl­phospho­nium) diiodide di­chloro­methane disolvate, C_37_H_32_P_2_^2+^·2I^−^·2CH_2_Cl_2_ or [Ph_3_PCH_2_PPh_3_]I_2_·2CH_2_Cl_2_ (**I**), methyl­enebis(tri­phenyl­phospho­nium) bis­(tetra­iodo­borate), C_37_H_32_P_2_^2+^·2BI_4_^−^ or [Ph_3_PCH_2_PPh_3_](BI_4_)_2_ (**II**). Solvents are the source of the protons at the ylidic C atom. The P—C—P angle is 124.1 (2)° for (**I**) and 121.7 (3)° for (**II**), while the two P—C bond lengths are 1.804 (4) and 1.807 (5) Å in (**I**), and 1.817 (5) and 1.829 (5) Å in (**II**). In the crystal of (**I**), the protons of the central P—CH_2_—P C atom exhibit weak C—H⋯I hydrogen bonds with the respective anions. The anions in turn are linked to the di­chloro­methane solvent mol­ecules by C—H⋯I hydrogen bonds. In the crystal of (**II**), one of the BI_4_^−^ anions is linked to a phenyl H atom *via* a weak C—H⋯I hydrogen bond.

## Abstract 2

Reaction of BI_3_ with carbodi­phospho­rane, C(PPh_3_)_2_, gives a mixture of the dicationic compounds, [Ph_3_PCH_2_PPh_3_]I_2_·2CH_2_Cl_2_ and [Ph_3_PCH_2_PPh_3_](BI_4_)_2_. Solvents are the source of the protons at the ylidic C atom.

## Chemical context   

Carbodi­phospho­ranes, C(PH_3_)_2_, have been known since the early 1960s (Ramirez *et al.*, 1961 ▸), but recent theoretical and experimental investigations has revived inter­est in these compounds (Tay *et al.*, 2016 ▸; Dordevic *et al.*, 2016 ▸). Theoretical studies (Frenking & Tonner, 2009 ▸) show the presence of two lone pairs of electrons, σ and π, which can act both as σ- and π-donor substituents (Tay *et al.*, 2013 ▸). Herein, we report on the crystal structures of two dicationic carbodiphophorane species, *viz.* [Ph_3_PCH_2_PPh_3_]I_2_·2CH_2_Cl_2_, (**I**), and [Ph_3_PCH_2_PPh_3_](BI_4_)_2_, (**II**).
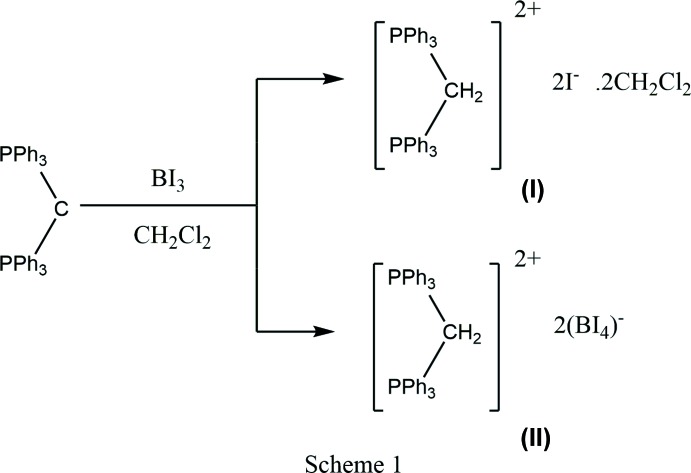


## Structural commentary   

**Figure 1:**
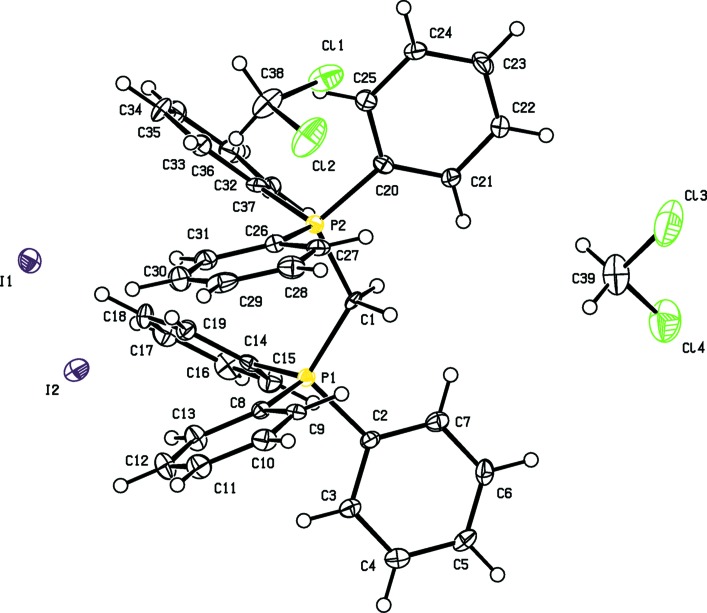
The mol­ecular structure of compound (**I**), showing the atom labelling and 40% probability displacement ellipsoids.

**Figure 2:**
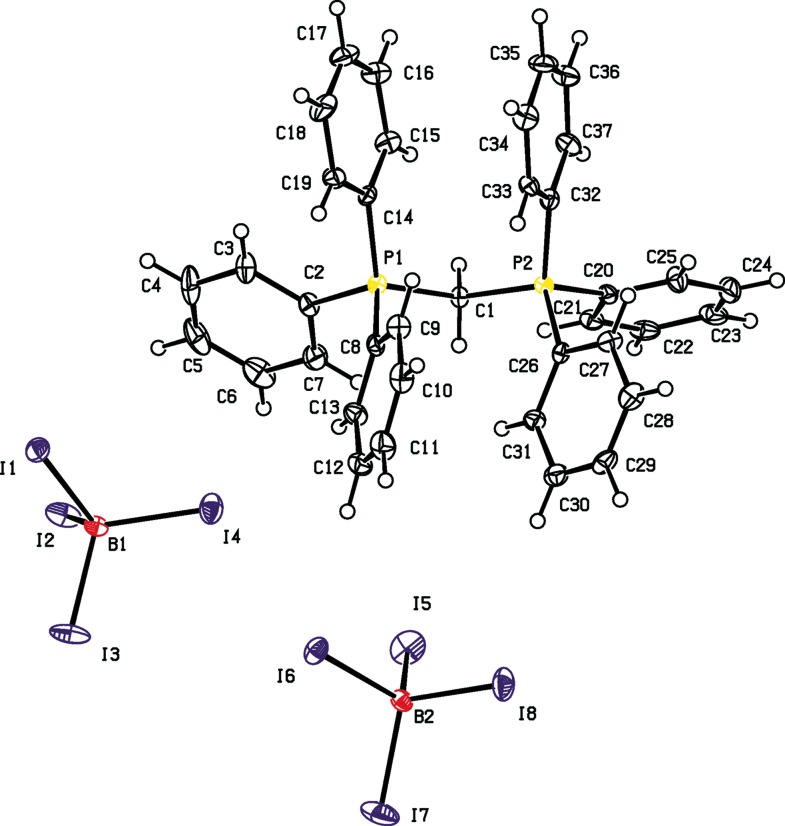
The mol­ecular structure of compound (**II**), showing the atom labelling and 40% probability displacement ellipsoids.

Compound [Ph_3_PCH_2_PPh_3_]I_2_, (**I**), crystallizes as a di­chloro­methane disolvate (Fig. 1 ▸), whereas compound (**II**) is not solvated (Fig. 2 ▸). For both compounds, the C2/P1/C1/P2/C20 fragment lies in a plane, as shown in Figs. 1 ▸ and 2 ▸, respectively, with the P1—C1—P2 angle being 124.1 (2)° for (**I**) and 121.7 (3)° for (**II**); see Tables 1 ▸ and 2 ▸. Such a conformation avoids any significant steric repulsion between the phenyl groups on the adjacent P atoms. The smaller value in compound (**II**) is attributed to decreased steric repulsion and/or an absence of electrostatic repulsion (Walker & Poli, 1989 ▸). The P—C bond lengths in compound (**I**) are slightly shorter than those in compound (**II**); see Tables 1 ▸ and 2 ▸. In (**II**), the BI_4_^−^ anions display regular tetra­hedral geometry, with I—B—I angles ranging from 108.1 (3) to 110.9 (3)°.

## Supra­molecular features   

**Figure 3:**
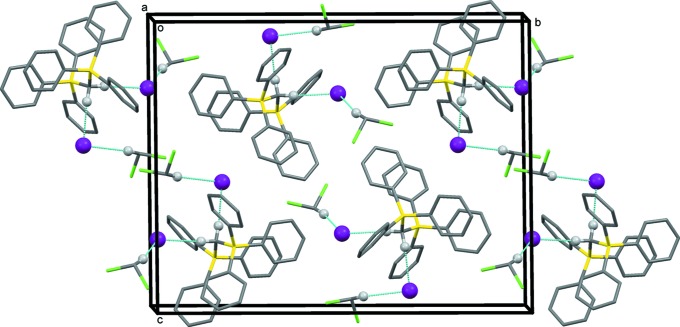
A view along the *a* axis of the crystal packing of compound (**I**). Only the H atoms (grey balls) participating in hydrogen bonding (dashed lines) have been included (see Table 3 ▸).

**Figure 4:**
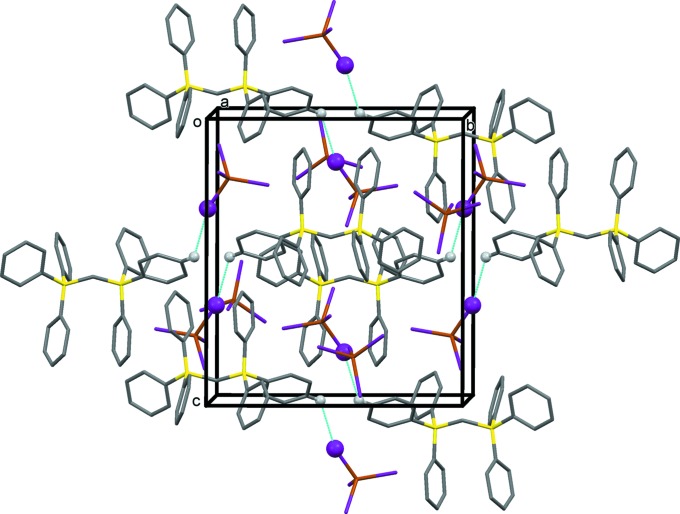
A view along the *a* axis of the crystal packing of compound (**II**). Only the H atom (grey ball) participating in hydrogen bonding (dashed lines) has been included (see Table 4 ▸).

In the crystal of (**I**), the iodide anion I1 forms weak hydrogen bonds with atoms H1*A* and H39*A*, while iodide anion I2 forms another pair of weak hydrogen bonds with atoms H1*B* and H38*B*, as shown in Table 3 ▸ and Fig. 3 ▸. In the crystal of (**II**), a single C—H⋯I hydrogen bond is observed linking an anion to the [Ph_3_PCH_2_PPh_3_]^2+^ unit (Table 4 ▸ and Fig. 4 ▸).

## Database survey   

A search of the Cambridge Structural Database (Version 5.38, last update May 2016; Groom *et al.*, 2016 ▸) revealed eight reported structures of the dicationic species, which all show similar conformations. In these eight structures, the P—C—P angle varies from *ca* 120.89 to 123.35°, while the P—C bond lengths vary from *ca* 1.802 to 1.833 Å. The smallest P—C—P angle and the shortest P—C bond length, *ca* 120.89° and 1.802 Å, respectively, are observed in methyl­enebis(tri­phenyl­phospho­nium) dichloride 1,2-di­meth­oxy­ethane monosolvate (CSD refcode CADZUE; Petz *et al.*, 2011 ▸). While one of the largest P—C—P angles (*ca* 123.11°) and longest P—C bond lengths (*ca* 1.825 Å) were observed for methyl­enebis(tri­phenyl­phospho­nium) bis­(tetra­chloro­indium) di­chloro­methane monosolvate (CIYGIB; Petz *et al.*, 2008 ▸). Inter­estingly, in compound (**I**), the P—C bond lengths are short [1.804 (4) and 1.807 (5) Å], while the P—C—P angle [124.1 (2)°] is one of the largest observed to date.

## Synthesis and crystallization   

(Ph_3_)_2_C (0.1 g, 0.19 mmol) and 1 equivalent of BI_3_ were mixed in *ca* 10 ml of DCM and left to stir overnight under inert conditions. The volume of the resulting solution was reduced to *ca* 3 ml and layered with *ca* 5 ml of hexane. A crop of crystals formed in a few days [yield 0.02 g, 4% based on (PPh_3_)_2_C, for (**I**) and 0.015 g, 5% based on (PPh_3_)_2_C, for (**II**)].

## Refinement   

Crystal data, data collection and structure refinement details are summarized in Table 5 ▸. The H atoms were included in calculated positions and treated as riding atoms, with C—H = 0.95–0.99 Å and *U*_iso_(H) = 1.2*U*_eq_(C). For both compounds, a small number of reflections were affected by the beam stop and were omitted from the final cycles of refinement.

## Supplementary Material

Crystal structure: contains datablock(s) Global, II, I. DOI: 10.1107/S2056989017010295/su5375sup1.cif

Structure factors: contains datablock(s) I. DOI: 10.1107/S2056989017010295/su5375Isup2.hkl

Structure factors: contains datablock(s) II. DOI: 10.1107/S2056989017010295/su5375IIsup4.hkl

CCDC references: 1533031, 1552112

Additional supporting information:  crystallographic information; 3D view; checkCIF report

## Figures and Tables

**Table 1:** Selected geometric parameters (Å, °) for (**I**)

C1—P2	1.804 (4)	C1—P1	1.807 (5)
			
P1—C1—P2	124.1 (2)		

**Table 2:** Selected geometric parameters (Å, °) for (**II**)

C1—P2	1.817 (5)	C1—P1	1.829 (5)
			
P1—C1—P2	121.7 (3)		

**Table 3:** Hydrogen-bond geometry (Å, °) for (**I**)

*D*—H⋯*A*	*D*—H	H⋯*A*	*D*⋯*A*	*D*—H⋯*A*
C1—H1*A*⋯I1^i^	0.99	2.81	3.802 (4)	175
C39—H39*A*⋯I1^i^	0.99	3.05	3.986 (7)	159
C1—H1*B*⋯I2^i^	0.99	2.83	3.813 (4)	175
C38—H38*B*⋯I2^ii^	0.99	2.88	3.848 (6)	166

Symmetry codes: (i) 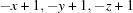; (ii) 
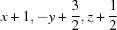
.

**Table 4:** Hydrogen-bond geometry (Å, °) for (**II**)

*D*—H⋯*A*	*D*—H	H⋯*A*	*D*⋯*A*	*D*—H⋯*A*
C23—H23⋯I1^i^	0.95	3.02	3.730 (7)	132

Symmetry code: (i) 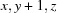.

**Table 5:** Experimental details

	(**I**)	(**II**)
Crystal data		
Chemical formula	C_37_H_32_P_2_^2+^·2I^−^·2CH_2_Cl_2_	C_37_H_32_P_2_^2+^·2BI_4_^−^
*M*_r_	962.22	1575.38
Crystal system, space group	Monoclinic, *P*2_1_/*c*	Monoclinic, *P*2_1_/*c*
Temperature (K)	153	153
*a*, *b*, *c* (Å)	9.7510 (13), 22.914 (3), 18.204 (2)	19.7878 (6), 14.3122 (3), 16.0646 (4)
β (°)	104.629 (2)	96.230 (1)
*V* (Å^3^)	3935.5 (9)	4522.7 (2)
*Z*	4	4
Radiation type	Mo *K*α	Mo *K*α
μ (mm^−1^)	1.98	5.58
Crystal size (mm)	0.14 × 0.12 × 0.06	0.14 × 0.12 × 0.08

Data collection		
Diffractometer	Bruker CCD area detector	Bruker CCD area detector
Absorption correction	Multi-scan (*SADABS*; Bruker, 2015 ▸)	Multi-scan (*SADABS*; Bruker, 2015 ▸)
*T*_min_, *T*_max_	0.74, 0.89	0.51, 0.66
No. of measured, independent and observed [*I* > 2σ(*I*)] reflections	18605, 7318, 5579	52611, 14486, 9439
*R*_int_	0.050	0.064
(sin θ/λ)_max_ (Å^−1^)	0.607	0.727

Refinement		
*R*[*F* ^2^ > 2σ(*F* ^2^)], *wR*(*F* ^2^), *S*	0.043, 0.083, 1.05	0.045, 0.124, 0.92
No. of reflections	7318	14486
No. of parameters	424	442
H-atom treatment	H-atom parameters constrained	H-atom parameters constrained
Δρ_max_, Δρ_min_ (e Å^−3^)	0.65, −0.71	2.32, −2.11

Computer programs: *APEX3* and *SAINT* (Bruker, 2015 ▸), *SHELXT* (Sheldrick, 2015*a* ▸), *Mercury* (Macrae *et al.*, 2008 ▸), *SHELXL2014* (Sheldrick, 2015*b* ▸), *PLATON* (Spek, 2009 ▸) and *publCIF* (Westrip, 2010 ▸).

## supplementary crystallographic information

### Methylenebis(triphenylphosphonium) diiodide dichloromethane disolvate (I) Crystal data

**Table:**

C_37_H_32_P_2_^2+^·2I^−^·2CH_2_Cl_2_	*F*(000) = 1896
*M**_r_* = 962.22	*D*_x_ = 1.624 Mg m^−^^3^
Monoclinic, *P*2_1_/*c*	Mo *K*α radiation, λ = 0.71073 Å
*a* = 9.7510 (13) Å	Cell parameters from 3861 reflections
*b* = 22.914 (3) Å	θ = 2.8–24.4°
*c* = 18.204 (2) Å	µ = 1.98 mm^−^^1^
β = 104.629 (2)°	*T* = 153 K
*V* = 3935.5 (9) Å^3^	Plate, colourless
*Z* = 4	0.14 × 0.12 × 0.06 mm

### Methylenebis(triphenylphosphonium) diiodide dichloromethane disolvate (I) Data collection

**Table:**

CCD area detector diffractometer	7318 independent reflections
Radiation source: fine-focus sealed tube, Bruker KappaCCD	5579 reflections with *I* > 2σ(*I*)
Graphite monochromator	*R*_int_ = 0.050
Detector resolution: 8.3333 pixels mm^-1^	θ_max_ = 25.5°, θ_min_ = 2.7°
phi and ω scans	*h* = −11→11
Absorption correction: multi-scan (SADABS; Bruker, 2015)	*k* = −27→27
*T*_min_ = 0.74, *T*_max_ = 0.89	*l* = −22→19
18605 measured reflections	

### Methylenebis(triphenylphosphonium) diiodide dichloromethane disolvate (I) Refinement

**Table:**

Refinement on *F*^2^	0 restraints
Least-squares matrix: full	Hydrogen site location: inferred from neighbouring sites
*R*[*F*^2^ > 2σ(*F*^2^)] = 0.043	H-atom parameters constrained
*wR*(*F*^2^) = 0.083	*w* = 1/[σ^2^(*F*_o_^2^) + (0.0157*P*)^2^ + 9.1897*P*] where *P* = (*F*_o_^2^ + 2*F*_c_^2^)/3
*S* = 1.05	(Δ/σ)_max_ = 0.001
7318 reflections	Δρ_max_ = 0.65 e Å^−^^3^
424 parameters	Δρ_min_ = −0.71 e Å^−^^3^

### Methylenebis(triphenylphosphonium) diiodide dichloromethane disolvate (I) Special details

**Table:**

Geometry. All esds (except the esd in the dihedral angle between two l.s. planes) are estimated using the full covariance matrix. The cell esds are taken into account individually in the estimation of esds in distances, angles and torsion angles; correlations between esds in cell parameters are only used when they are defined by crystal symmetry. An approximate (isotropic) treatment of cell esds is used for estimating esds involving l.s. planes.
Refinement. (I: reflections 0 0 2, 1 0 0, 0 2 1, 1 1 0, -1 1 1, 0 2 0, 1 1 1, -1 2 4, -1 2 1 and 0 1 1, were affected by the beam stop and omitted from the final cycles of refinement.

### Methylenebis(triphenylphosphonium) diiodide dichloromethane disolvate (I) Fractional atomic coordinates and isotropic or equivalent isotropic displacement parameters (Å^2^)

**Table:**

	*x*	*y*	*z*	*U*_iso_*/*U*_eq_	
C1	0.5915 (5)	0.66498 (19)	0.7366 (2)	0.0139 (10)	
H1A	0.5834	0.6725	0.7889	0.017*	
H1B	0.6265	0.6245	0.7362	0.017*	
C2	0.3140 (5)	0.6175 (2)	0.7224 (3)	0.0179 (11)	
C3	0.1671 (5)	0.6199 (2)	0.6984 (3)	0.0233 (12)	
H3	0.1225	0.6458	0.6588	0.028*	
C4	0.0859 (6)	0.5846 (2)	0.7323 (3)	0.0278 (13)	
H4	−0.0146	0.5861	0.716	0.033*	
C5	0.1509 (6)	0.5470 (2)	0.7900 (3)	0.0262 (13)	
H5	0.095	0.5231	0.8137	0.031*	
C6	0.2969 (6)	0.5441 (2)	0.8133 (3)	0.0247 (12)	
H6	0.3414	0.5181	0.8528	0.03*	
C7	0.3787 (6)	0.5791 (2)	0.7789 (3)	0.0221 (12)	
H7	0.4791	0.5767	0.7943	0.026*	
C8	0.3361 (5)	0.73662 (19)	0.6724 (3)	0.0160 (11)	
C9	0.3560 (5)	0.7676 (2)	0.7407 (3)	0.0196 (11)	
H9	0.4129	0.7519	0.7866	0.024*	
C10	0.2921 (5)	0.8211 (2)	0.7402 (3)	0.0243 (12)	
H10	0.3057	0.8423	0.7863	0.029*	
C11	0.2087 (6)	0.8445 (2)	0.6744 (3)	0.0273 (13)	
H11	0.1668	0.8819	0.6749	0.033*	
C12	0.1868 (6)	0.8138 (2)	0.6085 (3)	0.0297 (13)	
H12	0.1274	0.8295	0.5632	0.036*	
C13	0.2501 (6)	0.7597 (2)	0.6065 (3)	0.0249 (12)	
H13	0.2344	0.7387	0.5602	0.03*	
C14	0.4079 (5)	0.6383 (2)	0.5839 (3)	0.0178 (11)	
C15	0.3739 (6)	0.5799 (2)	0.5665 (3)	0.0265 (13)	
H15	0.3481	0.5552	0.6028	0.032*	
C16	0.3778 (6)	0.5583 (2)	0.4965 (3)	0.0296 (13)	
H16	0.354	0.5186	0.4845	0.036*	
C17	0.4160 (6)	0.5938 (2)	0.4434 (3)	0.0292 (13)	
H17	0.4191	0.5784	0.3954	0.035*	
C18	0.4496 (6)	0.6514 (2)	0.4606 (3)	0.0262 (13)	
H18	0.475	0.6757	0.4238	0.031*	
C19	0.4469 (5)	0.6746 (2)	0.5305 (3)	0.0202 (11)	
H19	0.471	0.7143	0.5421	0.024*	
C20	0.8846 (5)	0.6956 (2)	0.7980 (3)	0.0176 (11)	
C21	0.8860 (5)	0.6523 (2)	0.8515 (3)	0.0212 (11)	
H21	0.8018	0.6315	0.8516	0.025*	
C22	1.0114 (6)	0.6397 (2)	0.9046 (3)	0.0249 (12)	
H22	1.0131	0.6097	0.9408	0.03*	
C23	1.1341 (6)	0.6703 (2)	0.9056 (3)	0.0269 (13)	
H23	1.2197	0.6613	0.9423	0.032*	
C24	1.1314 (6)	0.7142 (2)	0.8528 (3)	0.0264 (12)	
H24	1.215	0.7357	0.8538	0.032*	
C25	1.0084 (6)	0.7266 (2)	0.7991 (3)	0.0256 (12)	
H25	1.0074	0.7564	0.7626	0.031*	
C26	0.6846 (5)	0.7867 (2)	0.7281 (3)	0.0172 (11)	
C27	0.7029 (5)	0.8101 (2)	0.8007 (3)	0.0226 (12)	
H27	0.7453	0.7875	0.8442	0.027*	
C28	0.6589 (6)	0.8667 (2)	0.8089 (3)	0.0292 (13)	
H28	0.6709	0.883	0.8581	0.035*	
C29	0.5976 (6)	0.8995 (2)	0.7454 (3)	0.0318 (14)	
H29	0.5678	0.9383	0.751	0.038*	
C30	0.5794 (6)	0.8761 (2)	0.6738 (3)	0.0315 (14)	
H30	0.5378	0.8992	0.6306	0.038*	
C31	0.6212 (5)	0.8193 (2)	0.6641 (3)	0.0238 (12)	
H31	0.6067	0.803	0.6147	0.029*	
C32	0.7769 (5)	0.6952 (2)	0.6357 (3)	0.0195 (11)	
C33	0.8345 (5)	0.7377 (2)	0.5967 (3)	0.0235 (12)	
H33	0.8408	0.7771	0.6132	0.028*	
C34	0.8822 (6)	0.7217 (2)	0.5340 (3)	0.0309 (14)	
H34	0.9199	0.7505	0.5071	0.037*	
C35	0.8754 (6)	0.6651 (2)	0.5106 (3)	0.0281 (13)	
H35	0.9083	0.6548	0.4675	0.034*	
C36	0.8204 (6)	0.6220 (2)	0.5494 (3)	0.0294 (13)	
H36	0.8179	0.5824	0.5336	0.035*	
C37	0.7700 (5)	0.6376 (2)	0.6108 (3)	0.0210 (11)	
H37	0.73	0.6087	0.6364	0.025*	
C38	0.9861 (7)	0.9392 (3)	0.8330 (3)	0.0417 (16)	
H38A	0.899	0.9367	0.791	0.05*	
H38B	1.0557	0.9634	0.8154	0.05*	
C39	0.7581 (8)	0.5313 (3)	0.9713 (3)	0.0547 (19)	
H39A	0.6844	0.562	0.9607	0.066*	
H39B	0.7995	0.5288	0.927	0.066*	
Cl1	1.05582 (17)	0.86895 (7)	0.85465 (9)	0.0467 (4)	
Cl2	0.9455 (2)	0.97306 (7)	0.91165 (10)	0.0580 (5)	
Cl3	0.8912 (3)	0.55037 (10)	1.05242 (10)	0.0822 (7)	
Cl4	0.6801 (2)	0.46429 (8)	0.98340 (10)	0.0677 (6)	
I1	0.44524 (4)	0.31630 (2)	0.06172 (2)	0.02704 (10)	
I2	0.28004 (4)	0.49269 (2)	0.25393 (2)	0.02568 (10)	
P1	0.41286 (14)	0.66527 (5)	0.67665 (7)	0.0155 (3)	
P2	0.73153 (14)	0.71173 (5)	0.72243 (7)	0.0155 (3)	

### Methylenebis(triphenylphosphonium) diiodide dichloromethane disolvate (I) Atomic displacement parameters (Å^2^)

**Table:**

	*U*^11^	*U*^22^	*U*^33^	*U*^12^	*U*^13^	*U*^23^
C1	0.018 (3)	0.015 (2)	0.012 (2)	0.000 (2)	0.011 (2)	−0.0007 (18)
C2	0.016 (3)	0.021 (3)	0.018 (3)	−0.003 (2)	0.007 (2)	0.000 (2)
C3	0.019 (3)	0.027 (3)	0.026 (3)	0.001 (2)	0.010 (3)	0.004 (2)
C4	0.020 (3)	0.033 (3)	0.032 (3)	−0.004 (2)	0.007 (3)	0.002 (2)
C5	0.028 (3)	0.025 (3)	0.031 (3)	−0.006 (2)	0.016 (3)	0.003 (2)
C6	0.033 (3)	0.027 (3)	0.016 (3)	0.004 (2)	0.007 (3)	0.004 (2)
C7	0.021 (3)	0.025 (3)	0.023 (3)	0.000 (2)	0.010 (3)	−0.003 (2)
C8	0.015 (3)	0.014 (2)	0.019 (3)	−0.003 (2)	0.004 (2)	0.0011 (19)
C9	0.011 (3)	0.027 (3)	0.020 (3)	0.000 (2)	0.003 (2)	0.002 (2)
C10	0.023 (3)	0.029 (3)	0.022 (3)	0.000 (2)	0.007 (3)	−0.009 (2)
C11	0.029 (3)	0.020 (3)	0.034 (3)	0.006 (2)	0.010 (3)	0.000 (2)
C12	0.031 (3)	0.027 (3)	0.028 (3)	0.007 (3)	0.001 (3)	0.007 (2)
C13	0.030 (3)	0.026 (3)	0.017 (3)	0.006 (2)	0.004 (3)	−0.006 (2)
C14	0.011 (3)	0.022 (3)	0.017 (3)	−0.001 (2)	−0.002 (2)	−0.001 (2)
C15	0.028 (3)	0.023 (3)	0.028 (3)	−0.005 (2)	0.006 (3)	−0.002 (2)
C16	0.035 (4)	0.021 (3)	0.031 (3)	−0.002 (2)	0.004 (3)	−0.008 (2)
C17	0.031 (3)	0.037 (3)	0.019 (3)	0.002 (3)	0.004 (3)	−0.010 (2)
C18	0.029 (3)	0.040 (3)	0.010 (2)	0.000 (3)	0.005 (2)	0.005 (2)
C19	0.020 (3)	0.022 (3)	0.019 (3)	−0.003 (2)	0.005 (2)	0.001 (2)
C20	0.016 (3)	0.018 (2)	0.019 (3)	0.002 (2)	0.003 (2)	−0.002 (2)
C21	0.017 (3)	0.027 (3)	0.019 (3)	−0.004 (2)	0.004 (2)	0.003 (2)
C22	0.023 (3)	0.030 (3)	0.021 (3)	0.000 (2)	0.005 (3)	0.008 (2)
C23	0.021 (3)	0.038 (3)	0.018 (3)	0.003 (3)	−0.003 (2)	0.001 (2)
C24	0.016 (3)	0.039 (3)	0.025 (3)	−0.004 (2)	0.006 (3)	0.004 (2)
C25	0.021 (3)	0.032 (3)	0.022 (3)	−0.006 (2)	0.002 (3)	0.002 (2)
C26	0.016 (3)	0.017 (2)	0.019 (3)	−0.001 (2)	0.006 (2)	−0.001 (2)
C27	0.013 (3)	0.027 (3)	0.029 (3)	−0.002 (2)	0.006 (2)	0.001 (2)
C28	0.025 (3)	0.030 (3)	0.032 (3)	−0.004 (3)	0.006 (3)	−0.007 (2)
C29	0.025 (3)	0.020 (3)	0.056 (4)	−0.003 (2)	0.020 (3)	−0.004 (3)
C30	0.029 (3)	0.026 (3)	0.039 (3)	0.005 (3)	0.009 (3)	0.011 (3)
C31	0.020 (3)	0.024 (3)	0.027 (3)	−0.003 (2)	0.006 (3)	0.001 (2)
C32	0.009 (3)	0.028 (3)	0.021 (3)	−0.001 (2)	0.003 (2)	0.002 (2)
C33	0.021 (3)	0.028 (3)	0.021 (3)	−0.002 (2)	0.004 (2)	0.002 (2)
C34	0.031 (3)	0.043 (3)	0.024 (3)	−0.002 (3)	0.017 (3)	0.011 (3)
C35	0.026 (3)	0.045 (3)	0.015 (3)	0.008 (3)	0.008 (3)	0.003 (2)
C36	0.027 (3)	0.038 (3)	0.024 (3)	0.002 (3)	0.008 (3)	−0.007 (2)
C37	0.023 (3)	0.024 (3)	0.018 (3)	0.003 (2)	0.008 (2)	0.000 (2)
C38	0.050 (4)	0.043 (4)	0.042 (4)	−0.011 (3)	0.027 (4)	−0.002 (3)
C39	0.077 (6)	0.049 (4)	0.036 (4)	0.004 (4)	0.008 (4)	0.007 (3)
Cl1	0.0424 (10)	0.0460 (9)	0.0603 (10)	−0.0025 (8)	0.0290 (9)	−0.0043 (8)
Cl2	0.0904 (15)	0.0384 (9)	0.0600 (11)	−0.0085 (9)	0.0462 (11)	−0.0062 (8)
Cl3	0.1066 (18)	0.0915 (15)	0.0420 (10)	−0.0446 (14)	0.0070 (12)	0.0157 (10)
Cl4	0.0869 (16)	0.0624 (12)	0.0483 (10)	−0.0130 (11)	0.0067 (11)	0.0028 (9)
I1	0.0287 (2)	0.0339 (2)	0.01825 (17)	0.00212 (16)	0.00550 (16)	−0.00344 (15)
I2	0.0279 (2)	0.01925 (17)	0.0337 (2)	0.00037 (15)	0.01483 (17)	0.00000 (14)
P1	0.0156 (7)	0.0181 (6)	0.0130 (6)	−0.0011 (5)	0.0041 (6)	−0.0004 (5)
P2	0.0150 (7)	0.0172 (6)	0.0143 (6)	0.0007 (5)	0.0037 (6)	−0.0001 (5)

### Methylenebis(triphenylphosphonium) diiodide dichloromethane disolvate (I) Geometric parameters (Å, º)

**Table:**

C1—P2	1.804 (4)	C20—C25	1.396 (7)
C1—P1	1.807 (5)	C20—P2	1.794 (5)
C2—C7	1.380 (7)	C21—C22	1.384 (7)
C2—C3	1.389 (7)	C22—C23	1.383 (7)
C2—P1	1.795 (5)	C23—C24	1.387 (7)
C3—C4	1.383 (7)	C24—C25	1.372 (7)
C4—C5	1.381 (7)	C26—C31	1.391 (7)
C5—C6	1.381 (7)	C26—C27	1.396 (6)
C6—C7	1.387 (7)	C26—P2	1.787 (5)
C8—C13	1.383 (7)	C27—C28	1.385 (7)
C8—C9	1.402 (6)	C28—C29	1.382 (7)
C8—P1	1.792 (5)	C29—C30	1.377 (7)
C9—C10	1.373 (7)	C30—C31	1.390 (7)
C10—C11	1.376 (7)	C32—C37	1.391 (7)
C11—C12	1.360 (7)	C32—C33	1.403 (7)
C12—C13	1.388 (7)	C32—P2	1.786 (5)
C14—C15	1.396 (6)	C33—C34	1.385 (7)
C14—C19	1.403 (6)	C34—C35	1.361 (7)
C14—P1	1.787 (5)	C35—C36	1.397 (7)
C15—C16	1.377 (7)	C36—C37	1.376 (7)
C16—C17	1.384 (7)	C38—Cl1	1.754 (6)
C17—C18	1.377 (7)	C38—Cl2	1.760 (6)
C18—C19	1.387 (6)	C39—Cl4	1.752 (7)
C20—C21	1.388 (6)	C39—Cl3	1.758 (7)
			
P1—C1—P2	124.1 (2)	C24—C25—C20	120.1 (5)
C7—C2—C3	120.0 (5)	C31—C26—C27	120.6 (4)
C7—C2—P1	122.4 (4)	C31—C26—P2	122.3 (4)
C3—C2—P1	117.5 (4)	C27—C26—P2	116.9 (4)
C4—C3—C2	119.9 (5)	C28—C27—C26	119.6 (5)
C5—C4—C3	120.0 (5)	C29—C28—C27	119.9 (5)
C6—C5—C4	120.2 (5)	C30—C29—C28	120.4 (5)
C5—C6—C7	119.9 (5)	C29—C30—C31	120.9 (5)
C2—C7—C6	119.9 (5)	C30—C31—C26	118.7 (5)
C13—C8—C9	119.5 (4)	C37—C32—C33	119.1 (4)
C13—C8—P1	122.8 (4)	C37—C32—P2	119.2 (4)
C9—C8—P1	117.5 (4)	C33—C32—P2	121.4 (4)
C10—C9—C8	119.1 (5)	C34—C33—C32	119.6 (5)
C9—C10—C11	121.3 (5)	C35—C34—C33	120.6 (5)
C12—C11—C10	119.6 (5)	C34—C35—C36	120.6 (5)
C11—C12—C13	120.8 (5)	C37—C36—C35	119.2 (5)
C8—C13—C12	119.7 (4)	C36—C37—C32	120.8 (5)
C15—C14—C19	120.2 (4)	Cl1—C38—Cl2	112.1 (3)
C15—C14—P1	119.6 (4)	Cl4—C39—Cl3	111.4 (3)
C19—C14—P1	120.1 (4)	C14—P1—C8	111.3 (2)
C16—C15—C14	119.6 (5)	C14—P1—C2	109.7 (2)
C15—C16—C17	120.7 (5)	C8—P1—C2	107.9 (2)
C18—C17—C16	119.7 (5)	C14—P1—C1	111.2 (2)
C17—C18—C19	121.1 (5)	C8—P1—C1	111.0 (2)
C18—C19—C14	118.7 (5)	C2—P1—C1	105.5 (2)
C21—C20—C25	119.9 (5)	C32—P2—C26	112.3 (2)
C21—C20—P2	123.0 (4)	C32—P2—C20	106.8 (2)
C25—C20—P2	117.1 (4)	C26—P2—C20	109.2 (2)
C22—C21—C20	119.3 (5)	C32—P2—C1	111.5 (2)
C23—C22—C21	120.8 (5)	C26—P2—C1	110.4 (2)
C22—C23—C24	119.5 (5)	C20—P2—C1	106.3 (2)
C25—C24—C23	120.3 (5)		

### Methylenebis(triphenylphosphonium) diiodide dichloromethane disolvate (I) Hydrogen-bond geometry (Å, º)

**Table:**

*D*—H···*A*	*D*—H	H···*A*	*D*···*A*	*D*—H···*A*
C1—H1*A*···I1^i^	0.99	2.81	3.802 (4)	175
C39—H39*A*···I1^i^	0.99	3.05	3.986 (7)	159
C1—H1*B*···I2^i^	0.99	2.83	3.813 (4)	175
C38—H38*B*···I2^ii^	0.99	2.88	3.848 (6)	166

Symmetry codes: (i) −*x*+1, −*y*+1, −*z*+1; (ii) *x*+1, −*y*+3/2, *z*+1/2.

### Methylenebis(triphenylphosphonium) bis(tetraiodoborate) (II) Crystal data

**Table:**

C_37_H_32_P_2_^2+^·2BI_4_^−^	*F*(000) = 2872
*M**_r_* = 1575.38	*D*_x_ = 2.314 Mg m^−^^3^
Monoclinic, *P*2_1_/*c*	Mo *K*α radiation, λ = 0.71073 Å
*a* = 19.7878 (6) Å	Cell parameters from 7264 reflections
*b* = 14.3122 (3) Å	θ = 2.5–25.5°
*c* = 16.0646 (4) Å	µ = 5.58 mm^−^^1^
β = 96.230 (1)°	*T* = 153 K
*V* = 4522.7 (2) Å^3^	Block, colourless
*Z* = 4	0.14 × 0.12 × 0.08 mm

### Methylenebis(triphenylphosphonium) bis(tetraiodoborate) (II) Data collection

**Table:**

CCD area detector diffractometer	14486 independent reflections
Radiation source: fine-focus sealed tube, Bruker KappaCCD	9439 reflections with *I* > 2σ(*I*)
Graphite monochromator	*R*_int_ = 0.064
Detector resolution: 8.3333 pixels mm^-1^	θ_max_ = 31.1°, θ_min_ = 1.8°
phi and ω scans	*h* = −28→27
Absorption correction: multi-scan (SADABS; Bruker, 2015)	*k* = −20→20
*T*_min_ = 0.51, *T*_max_ = 0.66	*l* = −23→19
52611 measured reflections	

### Methylenebis(triphenylphosphonium) bis(tetraiodoborate) (II) Refinement

**Table:**

Refinement on *F*^2^	0 restraints
Least-squares matrix: full	Hydrogen site location: inferred from neighbouring sites
*R*[*F*^2^ > 2σ(*F*^2^)] = 0.045	H-atom parameters constrained
*wR*(*F*^2^) = 0.124	*w* = 1/[σ^2^(*F*_o_^2^) + (0.0618*P*)^2^ + 4.3616*P*] where *P* = (*F*_o_^2^ + 2*F*_c_^2^)/3
*S* = 0.92	(Δ/σ)_max_ = 0.001
14486 reflections	Δρ_max_ = 2.32 e Å^−^^3^
442 parameters	Δρ_min_ = −2.11 e Å^−^^3^

### Methylenebis(triphenylphosphonium) bis(tetraiodoborate) (II) Special details

**Table:**

Geometry. All esds (except the esd in the dihedral angle between two l.s. planes) are estimated using the full covariance matrix. The cell esds are taken into account individually in the estimation of esds in distances, angles and torsion angles; correlations between esds in cell parameters are only used when they are defined by crystal symmetry. An approximate (isotropic) treatment of cell esds is used for estimating esds involving l.s. planes.
Refinement. II: reflections 0 0 2, 2 0 0, 1 1 1, 2 1 0, -1 0 2, -2 1 1, -1 1 1 and 0 1 2, were affected by the beam stop and omitted from the final cycles of refinement.

### Methylenebis(triphenylphosphonium) bis(tetraiodoborate) (II) Fractional atomic coordinates and isotropic or equivalent isotropic displacement parameters (Å^2^)

**Table:**

	*x*	*y*	*z*	*U*_iso_*/*U*_eq_	
B1	0.4572 (3)	0.0659 (4)	0.2238 (4)	0.0196 (12)	
B2	0.9220 (3)	0.4312 (4)	0.1661 (4)	0.0209 (12)	
C1	0.6997 (3)	0.5283 (3)	0.5764 (3)	0.0163 (10)	
H1A	0.689	0.5285	0.5148	0.02*	
H1B	0.6557	0.5311	0.6003	0.02*	
C2	0.6780 (3)	0.3291 (3)	0.5616 (4)	0.0194 (11)	
C3	0.6740 (3)	0.2450 (4)	0.6055 (4)	0.0336 (15)	
H3	0.6999	0.236	0.6582	0.04*	
C4	0.6314 (4)	0.1752 (4)	0.5707 (6)	0.046 (2)	
H4	0.6286	0.1177	0.5996	0.056*	
C5	0.5937 (3)	0.1876 (5)	0.4963 (5)	0.0428 (19)	
H5	0.5651	0.1385	0.4736	0.051*	
C6	0.5958 (3)	0.2702 (5)	0.4526 (5)	0.0398 (16)	
H6	0.5683	0.2788	0.401	0.048*	
C7	0.6390 (3)	0.3410 (4)	0.4857 (4)	0.0321 (14)	
H7	0.6417	0.3979	0.4558	0.039*	
C8	0.8151 (3)	0.4054 (3)	0.5583 (4)	0.0186 (10)	
C9	0.8776 (3)	0.4303 (4)	0.6004 (4)	0.0238 (12)	
H9	0.8806	0.448	0.6576	0.029*	
C10	0.9357 (3)	0.4292 (4)	0.5589 (4)	0.0298 (14)	
H10	0.9785	0.4454	0.5879	0.036*	
C11	0.9310 (3)	0.4046 (4)	0.4758 (5)	0.0333 (15)	
H11	0.9707	0.4045	0.4474	0.04*	
C12	0.8694 (3)	0.3799 (4)	0.4332 (4)	0.0287 (13)	
H12	0.8668	0.3633	0.3757	0.034*	
C13	0.8110 (3)	0.3791 (4)	0.4739 (4)	0.0241 (12)	
H13	0.7687	0.361	0.4448	0.029*	
C14	0.7477 (3)	0.4003 (3)	0.7157 (3)	0.0183 (10)	
C15	0.6960 (3)	0.4326 (4)	0.7618 (4)	0.0249 (12)	
H15	0.6581	0.4654	0.7347	0.03*	
C16	0.7009 (3)	0.4160 (4)	0.8470 (4)	0.0309 (13)	
H16	0.666	0.4374	0.8785	0.037*	
C17	0.7562 (4)	0.3684 (4)	0.8869 (4)	0.0336 (14)	
H17	0.7594	0.3582	0.9456	0.04*	
C18	0.8066 (3)	0.3357 (4)	0.8414 (4)	0.0331 (14)	
H18	0.8443	0.3029	0.869	0.04*	
C19	0.8024 (3)	0.3507 (4)	0.7556 (4)	0.0237 (11)	
H19	0.8367	0.3271	0.7243	0.028*	
C20	0.6845 (3)	0.7276 (3)	0.5731 (3)	0.0177 (10)	
C21	0.6246 (3)	0.7120 (4)	0.5224 (3)	0.0209 (11)	
H21	0.6117	0.6502	0.5061	0.025*	
C22	0.5832 (3)	0.7867 (4)	0.4954 (4)	0.0282 (13)	
H22	0.542	0.7764	0.4604	0.034*	
C23	0.6024 (4)	0.8767 (4)	0.5201 (4)	0.0342 (15)	
H23	0.5738	0.9279	0.5026	0.041*	
C24	0.6626 (3)	0.8924 (4)	0.5695 (4)	0.0311 (14)	
H24	0.6757	0.9544	0.585	0.037*	
C25	0.7040 (3)	0.8184 (4)	0.5966 (4)	0.0258 (12)	
H25	0.7454	0.8292	0.6311	0.031*	
C26	0.8159 (2)	0.6531 (3)	0.5470 (3)	0.0156 (10)	
C27	0.8679 (3)	0.7133 (4)	0.5778 (4)	0.0222 (11)	
H27	0.8692	0.7374	0.6331	0.027*	
C28	0.9174 (3)	0.7379 (4)	0.5279 (4)	0.0294 (13)	
H28	0.9526	0.7796	0.5485	0.035*	
C29	0.9159 (3)	0.7025 (4)	0.4487 (4)	0.0264 (13)	
H29	0.9504	0.7199	0.415	0.032*	
C30	0.8655 (3)	0.6422 (4)	0.4165 (4)	0.0268 (13)	
H30	0.8655	0.6175	0.3617	0.032*	
C31	0.8144 (3)	0.6179 (4)	0.4658 (4)	0.0215 (11)	
H31	0.7786	0.5776	0.4442	0.026*	
C32	0.7668 (3)	0.6419 (3)	0.7154 (3)	0.0177 (10)	
C33	0.8287 (3)	0.6088 (3)	0.7527 (3)	0.0182 (10)	
H33	0.863	0.5903	0.719	0.022*	
C34	0.8404 (3)	0.6030 (4)	0.8390 (4)	0.0273 (12)	
H34	0.8825	0.5797	0.8647	0.033*	
C35	0.7913 (4)	0.6309 (4)	0.8873 (4)	0.0335 (14)	
H35	0.7993	0.6254	0.9465	0.04*	
C36	0.7303 (3)	0.6667 (4)	0.8515 (4)	0.0311 (14)	
H36	0.6974	0.6881	0.886	0.037*	
C37	0.7170 (3)	0.6715 (4)	0.7645 (4)	0.0254 (12)	
H37	0.6747	0.6946	0.7392	0.03*	
I1	0.50473 (2)	−0.01702 (3)	0.33197 (3)	0.02907 (10)	
I2	0.34522 (2)	0.03252 (3)	0.19998 (3)	0.03574 (11)	
I3	0.50580 (2)	0.02646 (4)	0.11044 (3)	0.03911 (12)	
I4	0.47021 (2)	0.21823 (3)	0.24780 (3)	0.03478 (11)	
I5	0.81419 (2)	0.44916 (3)	0.19353 (3)	0.04437 (13)	
I6	0.97031 (2)	0.31216 (3)	0.24298 (3)	0.02826 (10)	
I7	0.92629 (3)	0.40188 (4)	0.03197 (3)	0.04945 (14)	
I8	0.97951 (3)	0.56167 (3)	0.20371 (3)	0.04060 (12)	
P1	0.73738 (7)	0.41452 (9)	0.60508 (9)	0.0155 (3)	
P2	0.74413 (6)	0.63678 (8)	0.60451 (8)	0.0139 (2)	

### Methylenebis(triphenylphosphonium) bis(tetraiodoborate) (II) Atomic displacement parameters (Å^2^)

**Table:**

	*U*^11^	*U*^22^	*U*^33^	*U*^12^	*U*^13^	*U*^23^
B1	0.014 (3)	0.027 (3)	0.017 (3)	−0.003 (2)	−0.002 (2)	−0.004 (2)
B2	0.020 (3)	0.022 (3)	0.020 (3)	0.001 (2)	−0.001 (2)	−0.004 (2)
C1	0.013 (2)	0.015 (2)	0.020 (3)	0.0006 (18)	0.001 (2)	0.0000 (18)
C2	0.012 (2)	0.020 (2)	0.026 (3)	−0.0061 (18)	0.001 (2)	−0.004 (2)
C3	0.036 (4)	0.019 (3)	0.045 (4)	−0.006 (2)	0.002 (3)	−0.002 (3)
C4	0.047 (4)	0.017 (3)	0.076 (6)	−0.009 (3)	0.008 (4)	−0.006 (3)
C5	0.026 (3)	0.035 (3)	0.068 (5)	−0.011 (3)	0.009 (3)	−0.026 (3)
C6	0.028 (3)	0.050 (4)	0.039 (4)	−0.007 (3)	−0.007 (3)	−0.016 (3)
C7	0.036 (4)	0.029 (3)	0.030 (3)	−0.009 (3)	0.000 (3)	−0.002 (2)
C8	0.019 (3)	0.014 (2)	0.025 (3)	−0.0005 (19)	0.008 (2)	−0.0023 (19)
C9	0.018 (3)	0.026 (3)	0.028 (3)	0.000 (2)	0.000 (2)	0.000 (2)
C10	0.019 (3)	0.026 (3)	0.045 (4)	0.002 (2)	0.004 (3)	0.000 (3)
C11	0.026 (3)	0.029 (3)	0.047 (4)	0.003 (2)	0.013 (3)	−0.003 (3)
C12	0.033 (3)	0.027 (3)	0.028 (3)	0.000 (2)	0.012 (3)	−0.005 (2)
C13	0.022 (3)	0.026 (3)	0.024 (3)	−0.003 (2)	0.002 (2)	−0.005 (2)
C14	0.022 (3)	0.016 (2)	0.016 (3)	−0.0050 (19)	−0.001 (2)	0.0026 (18)
C15	0.021 (3)	0.031 (3)	0.023 (3)	−0.001 (2)	0.001 (2)	0.003 (2)
C16	0.029 (3)	0.042 (3)	0.023 (3)	−0.003 (3)	0.005 (3)	0.001 (3)
C17	0.041 (4)	0.040 (3)	0.020 (3)	−0.001 (3)	0.003 (3)	0.009 (3)
C18	0.036 (4)	0.034 (3)	0.027 (3)	0.003 (3)	−0.010 (3)	0.015 (3)
C19	0.027 (3)	0.024 (3)	0.019 (3)	0.002 (2)	−0.001 (2)	0.000 (2)
C20	0.019 (3)	0.017 (2)	0.018 (3)	0.0048 (18)	0.004 (2)	−0.0011 (19)
C21	0.019 (3)	0.026 (3)	0.017 (3)	0.004 (2)	−0.001 (2)	−0.001 (2)
C22	0.026 (3)	0.042 (3)	0.015 (3)	0.012 (3)	−0.001 (2)	0.003 (2)
C23	0.048 (4)	0.035 (3)	0.020 (3)	0.023 (3)	0.007 (3)	0.002 (2)
C24	0.044 (4)	0.021 (3)	0.027 (3)	0.011 (3)	−0.002 (3)	0.001 (2)
C25	0.027 (3)	0.020 (2)	0.029 (3)	0.004 (2)	−0.002 (2)	−0.002 (2)
C26	0.012 (2)	0.014 (2)	0.021 (3)	−0.0003 (17)	0.0027 (19)	0.0021 (18)
C27	0.017 (3)	0.030 (3)	0.019 (3)	−0.006 (2)	−0.001 (2)	0.001 (2)
C28	0.020 (3)	0.032 (3)	0.036 (4)	−0.006 (2)	0.004 (3)	0.004 (3)
C29	0.020 (3)	0.029 (3)	0.032 (3)	0.002 (2)	0.012 (2)	0.010 (2)
C30	0.030 (3)	0.026 (3)	0.025 (3)	0.008 (2)	0.008 (3)	0.005 (2)
C31	0.023 (3)	0.022 (2)	0.021 (3)	0.002 (2)	0.006 (2)	0.000 (2)
C32	0.017 (2)	0.017 (2)	0.019 (3)	−0.0017 (18)	−0.001 (2)	−0.0002 (19)
C33	0.017 (3)	0.019 (2)	0.018 (3)	−0.0006 (19)	−0.001 (2)	−0.0029 (19)
C34	0.022 (3)	0.031 (3)	0.026 (3)	−0.002 (2)	−0.009 (2)	0.001 (2)
C35	0.045 (4)	0.042 (3)	0.014 (3)	−0.008 (3)	0.005 (3)	−0.004 (2)
C36	0.036 (4)	0.036 (3)	0.023 (3)	0.001 (3)	0.010 (3)	−0.004 (2)
C37	0.022 (3)	0.028 (3)	0.026 (3)	0.004 (2)	0.006 (2)	−0.005 (2)
I1	0.0364 (2)	0.02556 (18)	0.0242 (2)	0.00250 (15)	−0.00166 (16)	0.00448 (15)
I2	0.01692 (19)	0.0617 (3)	0.0287 (2)	−0.00711 (17)	0.00315 (16)	−0.01338 (19)
I3	0.0213 (2)	0.0782 (3)	0.0185 (2)	0.0046 (2)	0.00497 (16)	−0.0110 (2)
I4	0.0394 (2)	0.02487 (18)	0.0370 (2)	−0.00048 (16)	−0.00956 (19)	0.00293 (16)
I5	0.0205 (2)	0.0557 (3)	0.0576 (3)	0.01384 (19)	0.0074 (2)	0.0110 (2)
I6	0.02340 (19)	0.02538 (17)	0.0370 (2)	0.00730 (14)	0.00765 (16)	0.00962 (15)
I7	0.0586 (3)	0.0673 (3)	0.0207 (2)	0.0183 (3)	−0.0038 (2)	−0.0119 (2)
I8	0.0512 (3)	0.02285 (19)	0.0443 (3)	−0.00971 (18)	−0.0105 (2)	−0.00049 (17)
P1	0.0129 (6)	0.0158 (6)	0.0175 (7)	−0.0006 (5)	−0.0006 (5)	0.0012 (5)
P2	0.0108 (6)	0.0163 (6)	0.0143 (6)	0.0015 (4)	0.0001 (5)	−0.0008 (5)

### Methylenebis(triphenylphosphonium) bis(tetraiodoborate) (II) Geometric parameters (Å, º)

**Table:**

B1—I3	2.224 (6)	C14—P1	1.779 (5)
B1—I4	2.224 (6)	C15—C16	1.382 (8)
B1—I1	2.226 (6)	C16—C17	1.386 (9)
B1—I2	2.258 (6)	C17—C18	1.380 (9)
B2—I7	2.206 (6)	C18—C19	1.389 (8)
B2—I8	2.236 (6)	C20—C21	1.382 (8)
B2—I5	2.241 (6)	C20—C25	1.396 (7)
B2—I6	2.255 (6)	C20—P2	1.791 (5)
C1—P2	1.817 (5)	C21—C22	1.388 (7)
C1—P1	1.829 (5)	C22—C23	1.388 (9)
C2—C7	1.382 (8)	C23—C24	1.376 (10)
C2—C3	1.402 (8)	C24—C25	1.381 (8)
C2—P1	1.784 (5)	C26—C27	1.390 (7)
C3—C4	1.385 (9)	C26—C31	1.395 (7)
C4—C5	1.350 (11)	C26—P2	1.792 (5)
C5—C6	1.377 (10)	C27—C28	1.378 (8)
C6—C7	1.394 (8)	C28—C29	1.368 (9)
C8—C9	1.390 (8)	C29—C30	1.376 (8)
C8—C13	1.400 (8)	C30—C31	1.394 (8)
C8—P1	1.788 (5)	C32—C33	1.389 (7)
C9—C10	1.389 (8)	C32—C37	1.392 (7)
C10—C11	1.374 (9)	C32—P2	1.791 (5)
C11—C12	1.379 (9)	C33—C34	1.384 (8)
C12—C13	1.387 (8)	C34—C35	1.367 (9)
C14—C19	1.391 (8)	C35—C36	1.377 (9)
C14—C15	1.405 (8)	C36—C37	1.396 (9)
			
I3—B1—I4	109.8 (3)	C21—C20—C25	120.3 (5)
I3—B1—I1	108.7 (3)	C21—C20—P2	123.1 (4)
I4—B1—I1	110.9 (3)	C25—C20—P2	116.5 (4)
I3—B1—I2	108.1 (3)	C20—C21—C22	120.0 (5)
I4—B1—I2	109.3 (3)	C23—C22—C21	119.4 (6)
I1—B1—I2	110.1 (3)	C24—C23—C22	120.7 (5)
I7—B2—I8	110.5 (3)	C23—C24—C25	120.2 (6)
I7—B2—I5	110.6 (3)	C24—C25—C20	119.4 (6)
I8—B2—I5	108.6 (2)	C27—C26—C31	119.8 (5)
I7—B2—I6	109.4 (2)	C27—C26—P2	119.6 (4)
I8—B2—I6	108.1 (3)	C31—C26—P2	119.8 (4)
I5—B2—I6	109.6 (3)	C28—C27—C26	119.7 (5)
P1—C1—P2	121.7 (3)	C29—C28—C27	120.2 (6)
C7—C2—C3	119.5 (5)	C28—C29—C30	121.6 (5)
C7—C2—P1	122.8 (4)	C29—C30—C31	118.9 (6)
C3—C2—P1	117.6 (5)	C30—C31—C26	119.8 (5)
C4—C3—C2	118.8 (7)	C33—C32—C37	120.3 (5)
C5—C4—C3	121.1 (7)	C33—C32—P2	121.9 (4)
C4—C5—C6	121.3 (6)	C37—C32—P2	117.4 (4)
C5—C6—C7	118.8 (7)	C34—C33—C32	119.7 (5)
C2—C7—C6	120.5 (6)	C35—C34—C33	120.0 (6)
C9—C8—C13	119.6 (5)	C34—C35—C36	121.0 (6)
C9—C8—P1	122.5 (4)	C35—C36—C37	119.9 (6)
C13—C8—P1	117.7 (4)	C32—C37—C36	119.0 (6)
C10—C9—C8	120.2 (6)	C14—P1—C2	108.2 (3)
C11—C10—C9	119.8 (6)	C14—P1—C8	113.6 (3)
C10—C11—C12	120.7 (6)	C2—P1—C8	110.2 (2)
C11—C12—C13	120.3 (6)	C14—P1—C1	110.7 (2)
C12—C13—C8	119.4 (5)	C2—P1—C1	106.2 (2)
C19—C14—C15	120.0 (5)	C8—P1—C1	107.8 (2)
C19—C14—P1	121.2 (4)	C32—P2—C20	109.7 (2)
C15—C14—P1	118.5 (4)	C32—P2—C26	112.4 (2)
C16—C15—C14	119.3 (6)	C20—P2—C26	107.2 (2)
C15—C16—C17	120.5 (6)	C32—P2—C1	110.1 (2)
C18—C17—C16	120.2 (6)	C20—P2—C1	105.3 (2)
C17—C18—C19	120.3 (6)	C26—P2—C1	111.9 (2)
C18—C19—C14	119.7 (5)		

### Methylenebis(triphenylphosphonium) bis(tetraiodoborate) (II) Hydrogen-bond geometry (Å, º)

**Table:**

*D*—H···*A*	*D*—H	H···*A*	*D*···*A*	*D*—H···*A*
C23—H23···I1^i^	0.95	3.02	3.730 (7)	132

Symmetry code: (i) *x*, *y*+1, *z*.
